# Supplementary material for: Integrative analysis of DNA replication origins and ORC-/MCM-binding sites in human cells reveals a lack of overlap
Source: eLife. 2024 Apr 3;12:RP89548. doi: 10.7554/eLife.89548 (PMC10990492; doi:10.7554/eLife.89548)
Supplement: MDAR checklist [file elife-89548-mdarchecklist1.docx]

Materials Design Analysis Reporting (MDAR) Checklist for Authors

The MDAR framework establishes a minimum set of requirements in transparent reporting applicable to studies in the life sciences (see Statement of Task: doi:10.31222/osf.oio/9sm4x.). The MDAR checklist is a tool for authors, editors and others seeking to adopt the MDAR framework for transparent reporting in manuscripts

and other outputs. Please refer to the MDAR Elaboration Document for additional context for the MDAR framework.

| **Antibodies** | **Yes (indicate where provided: page no/section/legend)** | **n/a** |
| --- | --- | --- |
| For commercial reagents, provide supplier name, catalogue number and RRID, if available |  | n/a |

| **Cell Materials** | **Yes (indicate where provided: page no/section/legend)** | **n/a** |
| --- | --- | --- |
| **Cell lines:** Provide species information, strain. Provide accession number in repository OR supplier name, catalog number, clone number, OR RRID |  | n/a |
| **Primary cultures:** Provide species, strain, sex of origin, genetic modification status. |  | n/a |

| **Experimental Animals** | **Yes (indicate where provided: page no/section/legend)** | **n/a** |
| --- | --- | --- |
| **Laboratory animals:** Provide species, strain, sex, age, genetic modification status. Provide accession number in repository OR supplier name, catalog number, clone number, OR RRID |  | n/a |
| **Animal observed in or captured from the field:**  Provide species, sex and age where possible |  | n/a |
| **Model organisms:** Provide Accession number in repository (where relevant) OR RRID |  | n/a |

| **Plants and microbes** | **Yes (indicate where provided: page no/section/legend)** | **n/a** |
| --- | --- | --- |
| **Plants:** provide species and strain, unique accession number if available, and source (including location for collected wild specimens) |  | n/a |
| **Microbes:** provide species and strain, unique accession number if available, and source |  | n/a |

| **Human research participants** | **Yes (indicate where provided: page no/section/legend)** | **n/a** |
| --- | --- | --- |
| Identify authority granting ethics approval (IRB or equivalent committee(s), provide reference number for approval. |  | n/a |
| Provide statement confirming informed consent obtained from study participants. |  | n/a |
| Report on age and sex for all study participants. |  | n/a |

| **Study protocol** | **Yes (indicate where provided: page no/section/legend)** | **n/a** |
| --- | --- | --- |
| For clinical trials, provide the trial registration number OR cite DOI in manuscript. |  | n/a |

| **Laboratory protocol** | **Yes (indicate where provided: page no/section/legend)** | **n/a** |
| --- | --- | --- |
| Provide DOI or other citation details if detailed step- by-step protocols are available. |  | n/a |

| **Experimental study design (statistics details)** | **Yes (indicate where provided: page no/section/legend)** | **n/a** |
| --- | --- | --- |
| State whether and how the following have been done, or if they were not carried out |  |  |
| Sample size determination |  | n/a |
| Randomization |  | n/a |
| Blinding |  | n/a |
| inclusion/exclusion criteria |  | n/a |

| **Sample definition and in-laboratory replication** | **Yes (indicate where provided: page no/section/legend)** | **n/a** |
| --- | --- | --- |
| State number of times the experiment was replicated in laboratory |  | n/a |
| Define whether data describe technical or biological replicates |  | n/a |

| **Ethics** | **Yes (indicate where provided: page no/section/legend)** | **n/a** |
| --- | --- | --- |
| Studies involving human participants: State details of authority granting ethics approval (IRB or equivalent committee(s), provide reference number for approval. |  | n/a |
| Studies involving experimental animals: State details of authority granting ethics approval (IRB or  equivalent committee(s), provide reference number for approval. |  | n/a |
| Studies involving specimen and field samples: State if relevant permits obtained, provide details of authority approving study; if none were required, explain why. |  | n/a |

| **Dual Use Research of Concern (DURC)** | **Yes (indicate where provided: page no/section/legend)** | **n/a** |
| --- | --- | --- |
| If study is subject to dual use research of concern, state the authority granting approval and reference number for the regulatory approval |  | n/a |

| **Attrition** | **Yes (indicate where provided: page no/section/legend)** | **n/a** |
| --- | --- | --- |
| State if sample or data point from the analysis is excluded, and whether the criteria for exclusion were determined and specified in advance. |  | n/a |

| **Statistics** | **Yes (indicate where provided: page no/section/legend)** | **n/a** |
| --- | --- | --- |
| Describe statistical tests used and justify choice of tests. | The Odds ratio obtained from two-tailed Fisher’s exact test was used as the enrichment score for each peak file (Fig . 2j) |  |

| **Data availability** | **Yes (indicate where provided: page no/section/legend)** | **n/a** |
| --- | --- | --- |
| State whether newly created datasets are available, including protocols for access or restriction on access. |  | The current manuscript is a computational study, so no data have been generated for this manuscript. |
| If data are publicly available, provide accession number in repository or DOI or URL. |  | n/a |
| If publicly available data are reused, provide accession number in repository or DOI or URL, where possible. | SNS-seq raw data is generated from several papers and is accessible in GSE101515, GSE114703, GSE128477, GSE134988, GSE172417, GSE184353, GSE186675. Repli-seq defined origins is generated from UW Encode group and is accessible in GSE34399. Buble-seq raw data is accessible in GSE38809. OK-seq defined Initiation Zones is accessible in PRJEB25180 and https://github.com/CL-CHEN-Lab/OK-Seq/tree/master/published_results. Rerep-seq raw data is accessible in GSE143572. (Supplementary table 1)  G4(G-quadraplex) predicted motif sites are obtained from a published G4 motif predictor: G4Hunter (Bedrat et al. 2016). The 1,444,095 G4 motif coordinates is provided by Laurent. |  |

| **Code availability** | **Yes (indicate where provided: page no/section/legend)** | **n/a** |
| --- | --- | --- |
| For all newly generated code and software essential for replicating the main findings of the study: |  |  |
| State whether the code or software is available. | From line 658 to line 784. (Method section) |  |
| If code is publicly available, provide accession number in repository, or DOI or URL. | The source code and scripts for all data analysis are available at <https://github.com/tmx1228/Replication-Origins.git> |  |

| **Adherence to community standards** | **Yes (indicate where provided: page no/section/legend)** | **n/a** |
| --- | --- | --- |
| MDAR framework recommends adoption of discipline-specific guidelines, established and endorsed through community initiatives. Journals have their own policy about requiring specific guidelines and recommendations to complement MDAR. |  |  |
| State if relevant guidelines (eg., ICMJE, MIBBI, ARRIVE) have been followed, and whether a checklist (eg., CONSORT, PRISMA, ARRIVE) is provided with the manuscript. |  | n/a |
